# The Prognostic Value of Biomarkers in Non-ST-Elevation Acute Coronary Syndrome Patients that are Treated by an Early Invasive Strategy: Insights from the OPTIMA-2 Trial

**DOI:** 10.31083/j.rcm2404117

**Published:** 2023-04-18

**Authors:** Nick D. Fagel, Maarten A. Vink, Antonius A.C.M. Heestermans, Robert K. Riezebos

**Affiliations:** ^1^Heart Center, OLVG Hospital, 1091 AC Amsterdam, The Netherlands; ^2^Heart Center, NWZ Hospital, 1815 JD Alkmaar, The Netherlands

**Keywords:** acute coronary syndrome, timing, treatment strategy, biomarkers

## Abstract

**Background:**

Patients with non-ST-elevation acute coronary syndrome (NSTE-ACS) consists of a heterogenic population and improvement in identification of a specific risk profile is needed. In this study we aimed to obtain better insight in the role of different biomarkers for patients undergoing a routine invasive diagnostic strategy within 24 hours after admission.

**Methods:**

An Immediate or Early Invasive Strategy in Non-ST-Elevation Acute Coronary Syndrome (OPTIMA-2) study was a randomized controlled prospective open-label multicentre trial, randomizing NSTE-ACS patients. An invasive strategy was either immediate (<3 hours) or early (12–24 hours). Peak high-sensitive TroponinT (hsTropT) value was determined within the first 48 hours of admission. N-terminal proB-type natriuretic peptide (NTpro-BNP) and high-sensitivity C-reactive protein (hsCRP) values were determined at admission and at discharge. These biomarkers were then divided into tertiles and related to clinical outcomes up to one year. The relation between these biomarkers and myocardial function recovery established by echocardiography was analyzed as a secondary endpoint.

**Results:**

The OPTIMA-2 study included 249 patients. Overall, there was no significant increase in the risk of developing an adverse cardiovascular event in the first year if biomarker tertiles at admission were compared. However, mean NT-proBNP levels at admission were higher for patients that experienced all-cause death withing the first year (1.93 ± 0.49 vs 1.42 ± 0.58, *p* = 0.05). Also, peak hs-cTnT (232.0 ± 2846.0 vs 71.5 ± 1152.0, *p* = 0.06) values at baseline were higher in patients experiencing a myocardial infarction within 1-year. NT-proBNP levels at admission and at discharge correlated with recovery of the left ventricular (LV) function at 30 days (coefficient 0.021 (95% CI = 0.009–0.033) and coefficient 0.016 (95% CI = 0.005–0.027)).

**Conclusions:**

In NSTE-ACS patients treated by an early invasive strategy and administration of modern anticoagulant and antiplatelet therapy, multiple biomarker measurements during admission could not predict the occurrence of recurrent cardiovascular events within the first year of follow-up.

## 1. Introduction

Non-ST-elevation acute coronary syndrome (NSTE-ACS) consists of a heterogenic group of patients and can be considered a high-risk condition if not treated in a swift and appropriate way. In the risk assessment of patients with NSTE-ACS cardiac biomarkers form an integral part. The extent of ischemia, in-hospital risk for recurrent events as well as risk for future events could be further specified with the aid of cardiac biomarkers. Several studies have been performed targeting the optimal biomarker’s risk assessment in NSTE-ACS patients [1, 2, 3]. In particular, high-sensitive TroponinT (hsTropT) and creatine kinase myocardial-band (CK-MB) are well known and recommended by major guidelines [4, 5]. Biomarkers such as high-sensitivity C-reactive protein (hsCRP) and N-terminal proB-type natriuretic peptide (NTpro-BNP) are relatively easy to obtain at low-cost, but less used in clinical practice [6, 7, 8, 9, 10]. NT-proBNP is well known in the clinical evaluation of congestive heart failure (CHF). It is secreted by cardiomyocytes in a situation of increased wall stress. Higher serum levels of NT-proBNP are demonstrated in myocardial ischemia due to up-regulation of B-type natriuretic peptide (BNP) gene expression and correlate with the extent of coronary artery disease [11, 12]. In addition, hsCRP is a well-established marker for inflammation in coronary artery disease [13]. In unstable coronary artery disease, it is related to a long-term cardiovascular (CV) mortality risk [14].

The “An Immediate or Early Invasive Strategy in Non-ST-Elevation Acute Coronary Syndrome” (OPTIMA-2) trial has been published previously [15]. This study was primarily designed to observe the influence of timing of an immediate invasive strategy in NSTE-ACS patients in relation to infarct size and risk for adverse cardiovascular events. Patients were randomized either to a direct invasive strategy (<3 hours) or early strategy (12–24 hours). The study did not find a significant difference in the area under the curve for CK-MB or hsTropT, nor did it find statistically significant difference in clinical endpoints. Several biomarkers were measured at admission and at discharge. This study aims to assess the value of several pathophysiological diverse biomarkers regarding prognosis within this specific population of NSTE-ACS patients.

## 2. Materials and Methods

### 2.1 Study Design 

The OPTIMA-2 trial was a prospective, open-label, randomized controlled trial (Netherlands Trial Register identifier: NTR3861) performed at the OLVG hospital in Amsterdam, the Netherlands [15]. Subject with at least one high-risk criterium for NSTE-ACS who experienced chest pain in the 24 hours before admission were selected for study participation. We defined high-risk criteria were defined as: horizontal or downsloping ST depression more than 1mm in two contiguous leads, dynamic ST- or T-wave changes >1 mm in two contiguous leads, elevated hs-cTnT Essay >1× upper limit normal (ULN), (defined as >0.014 ug/L), a patient history of coronary artery disease, or at least two risk factors: diabetes mellitus, known hypertension, smoking, family history for ischemic heart disease, dyslipidaemia, peripheral artery disease or aged 60 and older. Major exclusion criteria were acute ST-elevation myocardial infarction (STEMI), refractory angina and hemodynamic instability. After inclusion, patients were assigned to immediate (<3 hours) or early (12–24 hours) coronary angiography (CAG).

### 2.2 Study Procedure

After admittance to the hospital hs-cTnT levels were measured every 6 hours for the first 48-hours. Peak hs-cTnT was determined as the highest value measured within this first 48-hours after admission [15]. Per protocol, the NTpro-BNP and hsCRP levels were determined at admission. The immunoassays for hsTroponin, NTpro-BNP and hsCRP were performed using the Roche Cobas 8000 system. The used assays during the complete study enrolment period were: Elecsys Troponin-T hs Roche, Elecsys proBNP hs Roche and Tina-quant C-reactive protein Roche (Roche Diagnostics Ltd., Rotkreuz, Switserland). The cutoff value based of the upper 99th percentile was 152 ng/L for NT-proBNP, 10 mg/L for hsCRP and 14 ng/L for hs-cTnT. Fresh serum samples were collected and analyzed in the hospital’s laboratory within one our of collection.

In case of significant abnormalities at initial CAG it was by operator’s decision whether to perform direct percutaneous coronary intervention (PCI) or to first discuss the patient in the hospital’s heart team and than decide the appropriate form of treatment: conservative, PCI or coronary artery bypass graft (CABG) surgery. Echocardiography was performed within 72-hours after hospitalization and at 30-day follow-up. Left ventricular function (LVF) was defined as left ventricular ejection fraction (LVEF) and global longitudinal strain (GLS), both expressed in percentage. LVF was determined for all patients by an experienced investigator, who was blinded for treatment allocation [16].

### 2.3 Endpoints

The primary endpoint of our current sub-analysis was clinical event rate at 1-year follow-up in relation to peak hs-cTnT, NT-proBNP and hsCRP at admission. Clinical events were defined as major adverse cardiac events (MACE): composite of all-cause death, myocardial infarction (MI) and unplanned revascularization; net adverse clinical events (NACE): composite of MACE and major bleeding (all bleeding according to the Bleeding Academic Research Consortium (BARC) scale types 3 through 5). Biomarkers were each sub-divided in tertiles before analysis. Secondary endpoints were the comparison of median/mean biomarker levels of patients experiencing a clinical event within the first year and the relation of biomarkers in comparison to recovery of left ventricular function, determined by the LVEF and GLS.

### 2.4 Study Follow-Up

Follow-up was in person at 30 days after discharge. At that point a follow-up echocardiogram was made. After 1-year follow-up by telephone was done. In case we were not able to reach a patient we contacted local authorities to find out whether this patient was still alive.

### 2.5 Statistical Analysis

Statistical analysis was done with SPSS (version 26.0 for Windows, SPSS, Inc., Chicago, IL, USA). The number of clinical events within the first year were compared for each tertile group for NT-proBNP, hsCRP and peak hs-cTnT. A comparison was made by using the chi-square test for categorical variables. In addition, an univariate survival analyses was performed for tertiles of NT-proBNP and hsCRP at admission in relation to MACE and NACE rate within the first year.

For the secondary analysis biomarker levels of patients experiencing a clinical event within the first year of follow-up were compared to biomarker levels of patients that were event-free in the first year. Baseline and biomarker findings were analyzed making use of a Student t test or Wilcoxon rank-sum test. After log-rank transformation, the mean NT-proBNP at admission and at discharge yielded normally distributed data and a Student *t* test was chosen as the most appropriate way of analysis. To assess the correlation for those specific biomarkers with (change in) LVF, we analyzed the data using univariate linear regression analysis. Beta coefficients were calculated with 95% CI. In case of statistically significant beta coefficients, relevant biomarkers were included in the multivariate regression model. Tests were 2-tailed and a value of *p *< 0.05 was considered statistically significant.

## 3. Results

### 3.1 Baseline Characteristics

Patients were included in the period of March 2013 and November 2018. We included a total of 249 patients in the OPTIMA-2 study [15]. Table 1 shows the baseline characteristics of the complete study population.

**Table 1.** **Baseline characteristics**.

		Subjects (n = 249)
Age, yrs		65.6 ± 11.1
Gender, male		181 (72.7)
Body mass index, kg/$m^{2}$		28.1 ± 5.6
Duration of chest pain before admission, hours (IQR)*		3.0 (1.3–9.0)
ST depression >0.1 mV or dynamic ST-segment changes		60 (24)
hsTropT >1ULN		185 (74)
Inclusion by clinical characteristics only		46 (18.5)
Nt-proBNP, ng/L admission		606 (1668)
Nt-proBNP, ng/L discharge		820 (1774)
hsCRP, mg/L admission		6.2 (13.5)
hsCRP, mg/L discharge		17.1 (31.5)
GRACE-risk score†		115.0 ± 28.5
Cardiac History		
	Previous MI	56 (22.5)
	Previous CABG	19 (7.6)
	Previous PCI	58 (23.3)
	Known congestive heart failure	2 (0.8)
Risk Factors		
	Hypertension	120 (48.2)
	Current smoking	92 (36.9)
	Diabetes	49 (19.7)
	Hypercholesterolemia	79 (31.7)
	Positive family history	68 (27.3)
	Peripheral artery disease	12 (4.8)
	Age over 60 years	140 (56.2)

Values are mean ± SD, or n (%) unless listed otherwise. * Values are median (IQR). † GRACE-risk score: in-hospital death. IQR, interquartile range; ULN, upper limit normal; Ntpro-BNP, N-terminal proB-type natriuretic peptide; hsCRP, high-sensitivity C-reactive protein; GRACE, global registry of acute coronary events; MI, myocardial infarction; CABG, coronary artery bypass graft; PCI, percutaneous coronary intervention; SD, standard deviation; hsTropT, high-sensitive TroponinT.

In total 72.7% of the patients were male. The mean age at the time of hospitalisation was 65.6 years (standard deviation (SD) ± 11.1). The mean GRACE risk score was 115.0 (SD ± 28.5). In total, 198 admission NT-proBNP (80%), 179 discharge NT-proBNP (72%), 245 admission hsCRP (98%) and 206 discharge hsCRP (83%) were available for analysis. The mean NT-proBNP at admission was 606 ng/L (SD ± 1668) and 820 ng/L (SD ± 1774) at discharge. At admission the mean hsCRP was 6.2 mg/L (SD ± 13.5) and 17.1 mg/L (SD ± 31.5) at discharge. Within the first 48-hours of admission mean peak hs-cTnT was 584 ng/L (SD ± 1274).

### 3.2 Endpoints

**Fig. 1.**
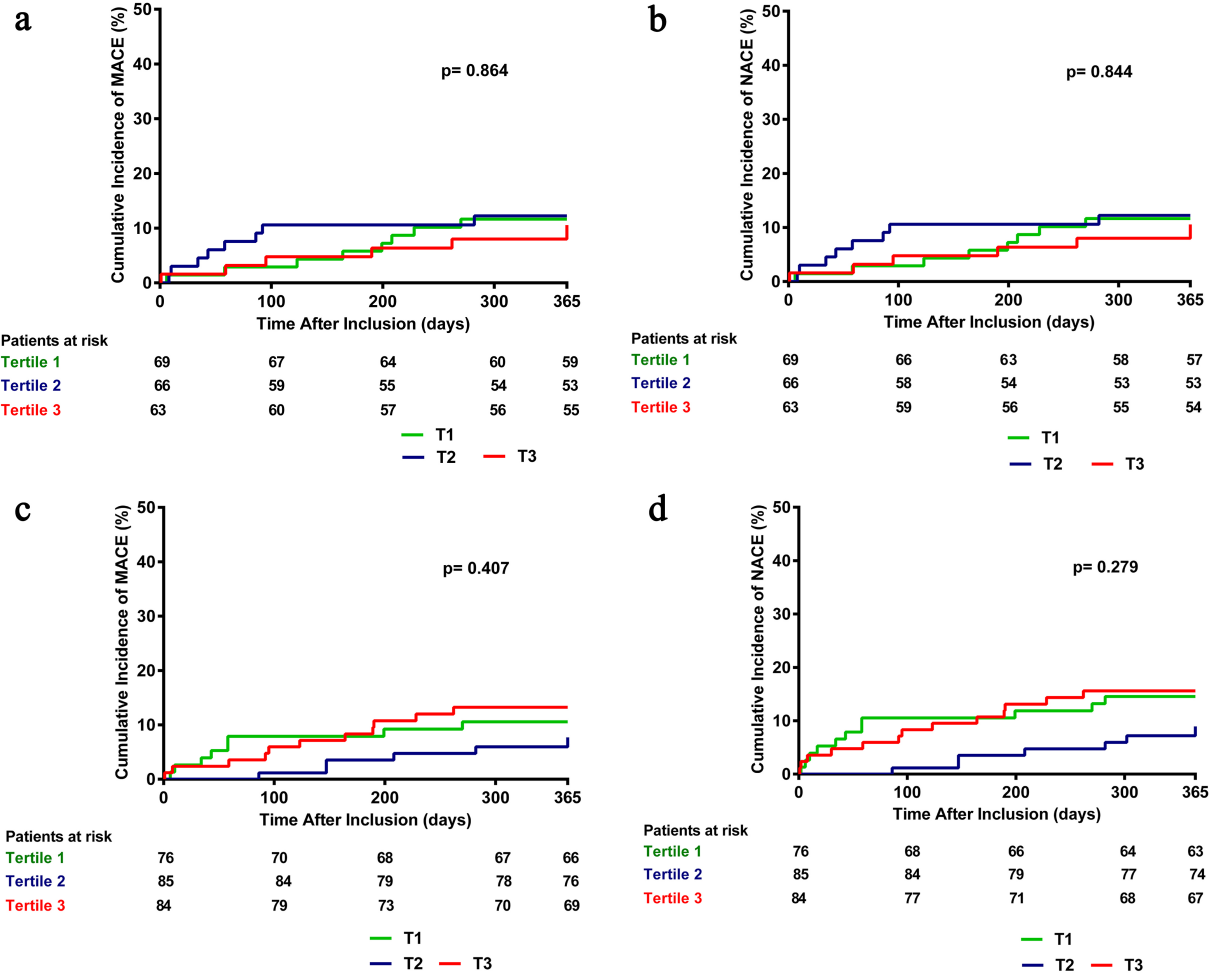
**Kaplan-Meier figures**. (a) NT-proBNP tertiles in relation to the occurence of MACE. (b) NT-proBNP tertiles in relation to the occurence of NACE. (c) hs-CRP tertiles in relation to the occurence of MACE. (d) hs-CRP tertiles in relation to the occurence of NACE. NTpro-BNP, N-terminal proB-type natriuretic peptide; MACE, major adverse cardiac events; NACE, net adverse clinical events; hsCRP, high-sensitivity C-reactive protein.

Biomarkers were divided into tertiles and compared for several clinical events at 1-year follow-up. The hs-cTnT levels were divided in the following tertiles: <37 ng/L, 38–288 ng/L and >289 . The NT-proBNP levels consisted of the following tertiles: <161 ng/L, 161–440 ng/L and >440 ng/L. For hsCRP the three sub-groups were: <1.5 mg/L, 1.5–4.0 mg/L and >4 mg/L. The comparison of cardiac biomarkers according to tertiles in relation to 1-year clinical events are shown in Table 2. In general, no significant difference in NT-proBNP, hsCRP or peak hs-cTnT tertile levels were observed. In addition, survival analysis showed similar outcome in MACE and NACE if the tertile groups were compared (Table 3, Fig. 1). The lack of difference between the tertile groups was observed for both NT-proBNP and hsCRP levels at admission.

**Table 2.** **Biomarkers in relation to 1-year clinical outcomes**.

	Peak hsTroponinT				NT-proBNP				hsCRP			
	T1 (n = 83)	T2 (n = 81)	T3 (n = 83)	*p*-value	T1 (n = 69)	T2 (n = 66)	T3 (n = 69)	*p*-value	T1 (n = 76)	T2 (n = 85)	T3 (n = 83)	*p*-value
Death (%)	2 (2)	3 (4)	2 (2)	0.82	0 (0)	2 (3)	3 (4)	0.21	1 (1)	3 (4)	3 (4)	0.80
Recurrent MI (%)	1 (1)	6 (7)	4 (5)	0.14	4 (6)	3 (5)	2 (3)	0.77	3 (4)	3 (4)	5 (6)	0.86
Recurrent Revascularization (%)	4 (5)	2 (2)	4 (5)	0.71	4 (6)	4 (7)	1 (1)	0.39	4 (5)	2 (2)	4 (5)	0.78
MACE (%)	5 (6)	11 (14)	9 (11)	0.24	8 (12)	8 (12)	6 (9)	0.89	8 (11)	6 (7)	11 (13)	0.60
NACE (%)	8 (10)	13 (16)	10 (12)	0.40	10 (14)	9 (14)	7 (10)	0.84	11 (14)	7 (8)	13 (16)	0.47

Values are number of patients (%). *p*-values were calculated from the chi-square test. NTpro-BNP, N-terminal proB-type natriuretic peptide; hsCRP, high-sensitivity C-reactive protein; T1, Tertile 1; T2, Tertile 2; T3, Tertile 3; MI, myocardial infarction; MACE, major adverse cardiac events; NACE, net adverse clinical events; hsTropT, high-sensitive TroponinT.

**Table 3.** **MACE and NACE survival analysis**.

Biomarker		MACE	Cox proportional-hazards regression	NACE	Cox proportional-hazards regression
		No (%)	HR (95% CI)	No (%)	HR (95% CI)
hsTroponinT					
	T1	5 (6)	1 (Ref)	8 (10)	1 (Ref)
	T2	11 (14)	2.56 (0.85–7.72)	13 (16)	1.87 (0.73–4.79)
	T3	9 (11)	1.90 (0.61–5.92)	10 (12)	1.28 (0.48–3.43)
NT-pro-BNP*					
	T1	8 (12)	1 (Ref)	10 (14)	1 (Ref)
	T2	8 (12)	1.05 (0.26–2.46)	9 (14)	0.93 (0.35–2.46)
	T3	6 (9)	0.80 (0.37–2.99)	7 (10)	0.74 (0.26–2.07)
hsCRP*					
	T1	8 (11)	1 (Ref)	11 (14)	1 (Ref)
	T2	6 (7)	0.65 (0.21–1.95)	7 (8)	0.53 (0.19–1.45)
	T3	11 (13)	1.30 (0.49–3.42)	13 (16)	1.01 (0.46–2.62)

* Values at admission. MACE, major adverse cardiac events; NACE, net adverse clinical events; NTpro-BNP, N-terminal proB-type natriuretic peptide; hsCRP, high-sensitivity C-reactive protein; HR, hazards ratio; CI, confidence interval; Ref, reference category; hsTropT, high-sensitive TroponinT.

If mean biomarker levels were compared, a trend was observed towards higher admission NT-proBNP levels in patients that deceased after the first year of follow-up in comparison to the patients that were alive (after log-rank transformation: 1.93 ± 0.49 vs 1.42 ± 0.58, *p* = 0.05). Another trend was observed regarding higher levels of hsCRP at discharge in patients that experienced a recurrent myocardial infarction in comparison to patients that did not (15.0 ± 54.9 vs 5.1 ± 29.7, *p* = 0.05) and for the peak hs-cTnT in patients that experienced an myocardial infarction (MI) within the first year versus those that did not (232.0 ± 2846.0 vs 71.5 ± 1152.0, *p* = 0.06) (Table 4).

**Table 4.** **Biomarkers and Clinical Events at 1-year follow-up**.

	Death			Recurrent Myocardial Infarction			Recurrent Revascularization			MACE			NACE		
	Yes (n = 7)	No (n = 238)	*p*-value	Yes (n = 11)	No (n = 238)	*p*-value	Yes (n = 10)	No (n = 239)	*p*-value	Yes (n = 25)	No (n = 224)	*p*-value	Yes (n = 31)	No (n = 218)	*p*-value
Peak hsTroponinT, ng/L* (SD)	78.0 (852)	76.5 (1285)	0.89	232.0 (2846)	71.5 (1152)	0.06	119.0 (1755)	78.0 (1247)	0.38	183.0 (2103)	70.5 (1141)	0.06	115.0 (1920)	71.5 (1154)	0.29
NT-proBNP, pmol/L† (admission; SD)	1.93 (0.49)	1.42 (0.58)	0.05	1.42 (0.64)	1.44 (0.58)	0.91	1.23 (0.47)	1.45 (0.59)	0.27	1.44 (0.60)	1.44 (0.58)	0.96	1.41 (0.65)	1.44 (0.58)	0.79
NT-proBNP, pmol/L† (discharge; SD)	2.02 (0.38)	1.51 (0.65)	0.12	1.54 (1.03)	1.52 (0.64)	0.94	1.38 (0.71)	1.52 (0.65)	0.56	1.59 (0.77)	1.51 (0.64)	0.65	1.47 (0.77)	1.52 (0.64)	0.76
hsCRP, mg/L (IQR)* (admission; SD)	2.20 (34.5)	2.35 (12.0)	0.32	2.90 (11.6)	2.30 (13.6)	0.51	2.70 (2.5)	2.30 (13.7)	0.67	2.90 (21.5)	2.30 (12.2)	0.62	2.50 (21.4)	2.30 (11.8)	0.82
hsCRP, mg/L (IQR)* (discharge; SD)	5.40 (63.5)	5.20 (30.5)	0.99	15.0 (54.9)	5.1 (29.7)	0.05	4.60 (41.1)	5.20 (31.0)	0.48	7.25 (52.4)	5.10 (27.5)	0.12	6.15 (49.2)	5.15 (27.8)	0.33

*Values are median (SD). *p* value was calculated by the Wilcoxon rank-sum test. † Values are mean (SD). Values after log-rank transformation. *p* value was calculated by student *T*-test. MACE, major adverse cardiac events; NACE, net adverse clinical events; NTpro-BNP, N-terminal proB-type natriuretic peptide; hsCRP, high-sensitivity C-reactive protein; IQR, interquartile range; SD, standard deviation; hsTropT, high-sensitive TroponinT.

Both, NT-proBNP and hs-CRP correlated significantly to the baseline echocardiogram GLS in a univariate regression analysis. At 30-day follow-up echocardiography, admission NT-proBNP (coefficient 0.021 (95% CI = 0.009–0.033), *p *≤ 0.01) and discharge NT-proBNP (coefficient 0.016 (95% CI = 0.005–0.027), *p *≤ 0.01) correlated significantly to improvement in LVEF. Also, the NT-proBNP level at admission was a predictor for the improvement in GLS determined at the follow-up echocardiogram in comparrison to baseline, with a coefficient of 0.007 (95% CI = 0.001–0.014, *p* = 0.03).

## 4. Discussion

The main finding of the present analysis: we did not observe a significantly increased risk of developing clinical events within the first year of follow up according to the height of admission peak hs-cTnT, NT-proBNP or hsCRP levels. In patients with recurrent MI or MACE within the first year we did observe a trend towards a higher peak hs-cTnT, and in patients that deceased withing one year higher NT-proBNP levels at admission were observed.

Previously, the relationship between different biomarkers and clinical outcomes in NSTE-ACS has been analyzed. An important study conducted by Omland *et al*. [7] showed the prognostic value of NT-proBNP in 609 patients with STEMI and NSTE-ACS. Median NT-proBNP levels in the sub-acute phase of these patients were significantly lower in long-term (median follow-up of 51 months) surviving patient compared to diseased patients (313 ng/L vs 922 ng/L, *p* = < 0.001) [7]. In line with these results, Sabatine *et al*. [1] published a multimarker approach as a sub-analysis of the Oral Glycoprotein IIb/IIIa Inhibition With Orbofiban in Patients With Unstable Coronary Syndromes (OPUS-TIMI 16) study. Baseline measurements were done of TroponinI (TnI), BNP and C-reactive protein (CRP) in 450 NSTE-ACS patients. Each additional elevated biomarker resulted in a doubling of the mortality risk at 10 months follow-up. In addition, an increased risk was found for the development of MI or chronic heart failure if one or more biomarkers was increased [1, 17]. A finding that is consistent with our study results regarding recurrent myocardial infarction within the first year. Furthermore, in a sub-analysis of the Metabolic Efficiency with Ranolazine for Less Ischemia in Non-ST Elevation Acute Coronary Syndrome (MERLIN TIMI-36) study, several biomarkers of 4352 NSTE-ACS patients were investigated (TnI, NT-proBNP, CRP and myeloperodixase (MPO)) by making use of a multivariable model. The risk of CV death increased in a stepwise fashion for each biomarker. Only NT-proBNP and TnI were associated in an independent way with CV death, after a mean follow-up to 343 days [18]. In line with this long-term prognostic value of biomarkers is a sub-analysis of the OPTIMA-1 trial, which showed an enhanced prediction for NSTE-ACS patients to evolve in an in-hospital MI if admission NT-proBNP was elevated (>30 ng/L) [19]. For this reason, NT-proBNP can be an important tool for the assessment of in-hospital risk and thereby provide a better estimation of timing of invasive management.

To the best of our knowledge, no data is yet available investigating different biomarkers as a predictor for adverse clinical outcomes in high-risk NSTE-ACS patients treated in accordance with the current clinical practice guidelines (i.e., both an early invasive strategy as well as potent P2Y12 inhibitors) [20]. The optimal adherence to the current guideline’s timing recommendation as well as optimal medical therapy could be an explanation for the difference in findings between our current study and some of the previous trials mentioned above. However, within the first year of follow-up, the event rate in our study was quite low so no definitive conclusions can be drawn by the current results. Furthermore, the correlation between NT-proBNP and recovery of LVEF is remarkable. It is plausible that patients with significant increase in NT-proBNP levels were those with a larger area of myocardial ischemia. Since both timing strategies, direct and early, resulted in short delay to revascularization, sufficient circumstances for recovery of myocardial function are expected. This might explain a relatively large difference between baseline and 30-day follow-up LVEF. Still, we should consider the current study results as hypothesis generating. It is key to obtain more data of cardiac biomarkers in relation to patient’s risk for NSTE-ACS patient treated according to the current treatment standard.

Several limitations should be mentioned regarding the current study. Firstly, the original OPTIMA-2 study was conducted in a randomized controlled setting and the power calculation was primarily done to show a difference in area under the curve (AUC) of CK-MB between patients treated in an urgent (<3 hours) versus an early (12–24 hours) timeframe. For this reason, the study was not powered to detect the relationship of biomarker levels and clinical outcome. Secondly, some aspects of the original OPTIMA-2 study could be considered as limitation to our current sub-analysis: as mentioned before, OPTIMA-2 had a long period of patient recruitment and was terminated early. Further, the study was conducted in a single center setting and we did not use an outside core laboratory. Thirdly, although routine blood sampling at admission and discharge was included in the study protocol a substantial amount of admission and discharge biomarkers was missing, which is a potential bias to our study. The main reasons for missing’s were lab or logistic errors.

## 5. Conclusions

Our results show that biomarker levels at and during admission for NSTE-ACS do not add value regarding risk for recurrent events when patients are treated by an early invasive strategy that includes modern anticoagulant and antiplatelet therapy. In addition, higher NT-proBNP and hsCRP levels do predict an increased left ventricular recovery at follow up, probably because of the lager area of myocardium at risk.

## Data Availability

The datasets generated and/or analyzed during the current study are not publicly available due to local rules an national laws but are available from the corresponding author on reasonable request.

## Abbreviations

CABG, coronary artery bypass graft; CI, confidence interval; CK-MB, creatine kinase-MB; hsCRP, high-sensitivity C-reactive protein; hs-cTnT, high-sensitivity troponin T; MACE, major adverse cardiac events; NACE, net adverse clinical events; NSTE-ACS, non-ST-elevation acute coronary syndrome; NSTEMI, non-ST-elevation myocardial infarction; NT-proBNP, N-terminal proB-type natriuretic peptide; STEMI, ST-elevation myocardial infarction; TnI, troponin I.
